# Free and Bound Phenolic Compound Content and Antioxidant Activity of Different Cultivated Blue Highland Barley Varieties from the Qinghai-Tibet Plateau

**DOI:** 10.3390/molecules23040879

**Published:** 2018-04-11

**Authors:** Xi-Juan Yang, Bin Dang, Ming-Tao Fan

**Affiliations:** 1 College of Food Science and Engineering, Northwest A & F University, Yangling 712100, China; yangxijuan1130@126.com; 2 State Key Laboratory of Plateau Ecology and Agriculture, Qinghai University, Xining, Qinghai 810016, China; dangbin811@tom.com; 3 Tibetan Plateau Key Laboratory of Agric-Product Processing, Qinghai Academy of Agricultural and Forestry Sciences, Xining, Qinghai 810016, China

**Keywords:** blue highland barley, variety, free phenolics, bound phenolics, antioxidant activity

## Abstract

In this study, the polyphenols composition and antioxidant properties of 12 blue highland barley varieties planted on the Qinghai-Tibet Plateau area were measured. The contents of the free, bound and total phenolic acids varied between 166.20–237.60, 170.10–240.75 and 336.29–453.94 mg of gallic acid equivalents per 100 g of dry weight (DW) blue highland barley grains, while the free and bound phenolic acids accounted for 50.09% and 49.91% of the total phenolic acids, respectively. The contents of the free, bound and total flavones varied among 20.61–25.59, 14.91–22.38 and 37.91–47.98 mg of catechin equivalents per 100 g of dry weight (DW) of blue highland barley grains, while the free and bound flavones accounted for 55.90% and 44.10% of the total flavones, respectively. The prominent phenolic compounds in the blue hulless barley grains were gallic acid, benzoic acid, syringic acid, 4-coumaric acid, naringenin, hesperidin, rutin, (+)-catechin and quercetin. Among these, protocatechuic acid, chlorogenic acid and (+)-catechin were the major phenolic compounds in the free phenolics extract. The most abundant bound phenolics were gallic acid, benzoic acid, syringic acid, 4-coumaric acid, benzoic acid, dimethoxybenzoic acid, naringenin, hesperidin, quercetin and rutin. The average contribution of the bound phenolic extract to the DPPH^•^ free radical scavenging capacity was higher than 86%, that of free phenolic extract to the ABTS^•+^ free radical scavenging capacity was higher than 79%, and that of free phenolic (53%) to the FRAP antioxidant activity was equivalent to that of the bound phenol extract (47%). In addition, the planting environment exerts a very important influence on the polyphenol composition, content and antioxidant activity of blue highland barley. The correlation analysis showed that 2,4-hydroxybenzoic acid and protocatechuic acid were the main contributors to the DPPH^•^ and ABTS^•+^ free radical scavenging capacity in the free phenolic extract, while chlorogenic acid, vanillic acid, ferulic acid and quercetin were the main contributors to the free radical scavenging capacity in the bound phenol extract. The study results show that the blue highland barley grains have rich phenolic compounds and high antioxidant activity, as well as significant varietal differences. The free and bound phenolic extracts in the blue hulless barley grains have an equivalent proportion in the total phenol, and co-exist in two forms. They can be used as a potential valuable source of natural antioxidants, and can aid in enhancing the development and daily consumption of foods relating to blue highland barley.

## 1. Introduction

Highland barley (*Hordeum vulgare* L. *var. nudum Hook. f*.) is a type of barley, also known as hulless barley, due to the separation of lemma from caryopsis in the grain, as well as its bareness of grain. It is a cultivated barley variety in botany. Highland barley grows on the Qinghai-Tibet Plateau at an elevation of 1400~4700 m, and it is the major crop in the Qinghai-Tibet Plateau region, as well as the main food relied on by farmers and herdsmen in the Tibetan area for sustenance [1,2]. Over the past decade, highland barley has attracted extensive attention due to its high levels of β-glucans and prominent hyperlipidemia, diabetes and anti-atherosclerosis effects [3,4]. However, there have been few reports on the phenolic compounds in highland barley. Recent studies have shown that phenolic compounds found in cereals are known for their beneficial antioxidative, antitumor, decreasing blood lipids and hypoglycemic of human health [5,6]. Moreover, the content of phenolic compounds in barley (0.1–0.4%) is much higher than other grains [7]. As is widely known, the Qinghai-Tibet Plateau region has the characteristics of extreme cold, hypoxia and intense UV radiation, and highland barley is a unique crop in this extreme environment. Therefore, research on the phenolic compounds and antioxidant activities of highland barley in the Qinghai-Tibet Plateau region may help to reveal the healthy core of highland barley, and guide its healthy consumption.

Colored highland barley, mainly including black, purple and blue highland barley, is a precious germplasm resource [8,9]. In the Qinghai-Tibet Plateau region, blue highland barley is the most widely used in production and consumption, thus it has a high research value. Research has proven that grain color and genotype may impact type, content and activity of phenolic compounds in barley [10,11,12]. It has also been reported that the contents of polyphenols and antioxidant activity in barley are affected by their varieties [13,14], growth locations, environmental conditions [13,15] and years of growth [13]. However, most reports have focused on hulled barley, while there have been few studies regarding naked barley on the Qinghai Tibet Plateau. At present, a small number of studies have reported the content of polyphenols and activity in highland barley. Gong [2] reported on these in different parts of highland barley. Liu [16] reported on the content of polyphenols and the main monomer composition in barley bran. Zhu [17] reported that enzymatic extraction could improve the content of phenolic acids and antioxidant activity in highland barley. However, there have been no reports regarding research on the phenolic compounds and their antioxidant activities in different varieties of blue highland barley from the Qinghai-Tibet Plateau region.

Most of the natural polyphenols in edible plants exist in a free or bound state form [18,19]. The structure or existing form of polyphenols determines their functional location in the gastrointestinal tract of the human body [20]. At present, most of the reports pertaining to polyphenols in highland barley have involved free phenolics [2,21,22], and there have been few reports regarding bound phenolics. Thus far, Xia [23] and Zhu [17] have reported on the free and bound polyphenols content and antioxidant activity in highland barley. However, the comparative studies on the composition of free and bound phenolics and their antioxidant activities in blue highland barley have rarely been reported. There have been no reports concerning the effects of varieties and regions on polyphenols of different forms and antioxidant activities of blue highland barley. Therefore, the objectives of the present study were as follows: (1) a comparison of the differences found in the contents and compositions of free and bound phenolics in several varieties of blue highland barley; (2) the measurement of the antioxidant activities of the free and bound phenolics; and (3) an examination of the relationship between the concentration of composition of free, bound phenolics and antioxidant activity in blue highland barley. This work has an instructive significance in improving our understanding of the health value of blue highland barley, as well as guiding its healthy consumption, and promoting its development and utilization.

## 2. Results and Discussion

### 2.1. Total Phenolic and Flavonoid Contents

The phenolic content in the blue highland barley was examined (Table 1). It was observed that the 12 blue highland barley samples showed differences in the contents of free, bound and total phenolics (*p* < 0.05). The total phenolic content was 336.21–453.94 mg of gallic acid eq./100 g DW, which was higher than that of corn (1.21–1.68 fold), wheat (2.71–3.60 fold), oat (3.11–4.20 fold) and rice (3.71–5.04 fold) [20]. Among them, Beiqing 2 had the highest content of total phenolics (453.94 ± 11.38 mg GAE/100 g DW), while Dulihuang had the lowest content (336.29 ± 5.42 mg GAE/100 g DW). The free and bound phenolic contents (170.11–240.75 mg of gallic acid eq./100 g DW) were lower than the free (410.1–551.2 mg of gallic acid eq./100 g DW) and bound phenolic contents (376.1–861.2 mg of gallic acid eq./100 g DW) in blue hulled barley reported by Abdel-Aal [12], and also lower than the content of free phenolics (407.52 mg GAE/100 g DW) in highland barley reported by Liu, H.L [16]. The difference in phenolic content was influenced by the varieties, cultivation environment, extracted solvents and extracted fractions. In our experiment, 80% acetone was used to extract free phenolics from highland barley, acid hydrolysis was used to extract the bound phenolic content. The extracted material was the whole grain of highland barley, while an 80% methanol and alkali process was used to extract the free and bound phenolic content from barley [12]. The polyphenols were extracted from highland barley bran by 60% acetone [16]. However, the content was higher than the content of free and bound phenolic in maize, wheat, oats and rice [20], thus suggesting that blue highland barley can be used as a good dietary source of phenolics. In about 46.7% of the tested highland barley varieties from Lhasa, Tibet and the Haibei region of Qinghai Province (Beiqing 8, Beijing 9, Zangqing 320, Zangqing 690 and Xunhua Lianglan), the free phenolic content was significantly higher than the bound phenolic content, and 53.3% of the highland barley varieties showed a higher bound phenolic content than free phenolic content. This result is different from previous reports which showed that 95% phenolic acid in the hulled barley exists in a combining form [12,24]. This signifies that the gene type and growth environment of blue highland barley are the critical points to influencing the free and bound phenolic contents in its grains. This is consistent with the report results of Griffiths and Welch [25].

In the present study, the free and bound phenolic contents accounted for 45.56–54.30% and 45.71–53.38% in the blue highland barley, respectively, with average values of 50.09% and 49.91%. Previous studies have shown that free and bound phenolic contents respectively exert their functional roles in the upper gastrointestinal tract and colon [20]. Therefore, according to the proportion of free and bound phenolic contents in the total phenolic compounds, it may be concluded that the consumption of blue-grained highland barley alone could be beneficial to the health of the human digestive tract and colon, and that its function is superior to that of other cereals.

In the blue highland barley samples, the free, bound and total flavonoid contents in the blue highland barley varieties were tested, and there were significant differences among the different varieties (*p* < 0.05) (Table 1). The content of total flavonoids was 37.91–47.98 mg CE/100 g, which was equivalent to the content in maize, wheat, oat and rice [20]. Among the blue grain highland barley varieties which were tested, Beiqing 2 (47.98 ± 2.00 mg CE /100 g DW) had the highest content of total flavonoids, and the content of Mennong 1 (37.93 ± 0.77 mg CE/100 g DW) was the lowest. In the present study, the content (20.61–25.59 mg CE/100 g) of free flavonoid in the tested varieties was significantly (*p <* 0.05) higher than the other bound flavonoid contents (14.91–22.38 mg/100 g). This is different from the existence form of flavonoid in common buckwheat [26], wheat, rice, corn and oat [20]. In addition, the numbers of flavonoids existing in a bound form in common buckwheat, wheat, rice, corn and oat are higher than those in a free form. However, similar to the existence form of flavone in tartary buckwheat [27] and black rice [28], the content of flavone existing in a free form is higher than that in a bound form. The contents of free and bound flavonoid respectively accounted for 50.71–65.27% and 34.71–49.33% in the content of the total flavonoids, with respective average values of 55.90% and 44.10%.

Among these, the content of free flavonoids in Zangqing 690 accounted for 65.27% of the total flavonoids, which is significantly higher than the other varieties tested (*p* < 0.05), thus signifying that edible Zangqing 690 can play a better protection role in the upper respiratory tract. Therefore, the comprehensive and accurate evaluation of the content of free and bound phenolic compounds in blue highland barley is of great value to guiding its development and utilization.

The 12 varieties of blue highland barley were divided according to the different locations. The average contents of free phenol, combined phenol, total phenol, free flavones, combined flavones and total flavones are shown in Table 2. It can be seen from the table that there is a significant difference (*p* < 0.05) between the average contents of the free, bound and total phenolics of the highland barley in the different locations, and highland barley samples showed differences in the contents of free, bound and total flavonoids (*p* < 0.05) which indicates that planting environment has a significant impact on the contents of the phenolics and flavonoids of highland barley. The contents of the free phenolic of the blue highland barley planted in Menyuan (Qinghai) and Lhasa (Tibet) have no significant difference, and were higher than that of the blue highland barley planted in Hezuo (Gansu). In addition, the contents (216.68 mg/100 g DW) of the bound and total phenolics of the blue highland barley planted in Qinghai are higher than those planted in Lhasa (Tibet) and Hezuo (Gansu), which is related to the fact that the content of phenolic can be improved by promoting the synthesis of plant phenylalanine ammonia lyase at low temperature [29]. Moreover, the average content of blue highland barley varieties in the three locations is higher than those of Croatia [30] (148.01–159.00 mg GAE/100 g DW) and Tunisian barley [31] (40.01–123.00 mg GAE/100 DW). In addition, the contents of free, bound and total flavonoids of the blue highland barley planted in Menyuan (Qinghai) and Lhasa (Tibet) show no significant difference, among which the content of free flavones of the blue highland barley planted in Lhasa (Tibet) (255.2 mg/100 g DW) is the highest. The content of bound (19.68 mg/100 g DW) and total flavonoids (42.39 mg/100 g DW) of the blue highland barley planted in Hezuo (Gansu) are the highest. This indicates that the content of phenolic of the blue highland barley planted in Menyuan (Qinghai) is higher, as is the content of flavonoid of the blue highland barley planted in Hezuo (Gansu). Aside from the influence of genotype of highland barley, the content may be related to the climate, temperature and other conditions of the planting environment of blue highland barley. Narwal [13] and other scholars have pointed out that the free phenolics of India barley are more greatly influenced by genotype, while the contents of bound and total phenolics have been affected more by the planting environment. Therefore, the content of blue highland barley is simultaneously influenced by both the genotype and environment of the varieties.

### 2.2. Composition and Content of Phenolic Compounds in Blue Highland Barley

**Figure 1:**
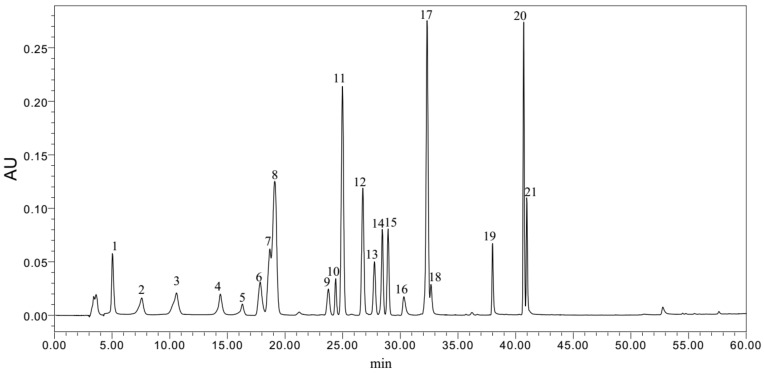
HPLC chromatogram of standards of phenolic compounds. (1) phloroglucinol; (2) gallic acid; (3) protocatechuic acid; (4) chlorogenic acid; (5) catechin; (6) 2,4-dihydroxybenzoic acid; (7) vanillic; (8) syringic acid; (9) *p*-coumaric; (10) rutin; (11) ferulic acid; (12) salicylic acid; (13) naringin; (14) hesperidin; (15) benzoic acid; (16) *o*-coumaric acid; (17) myricetin; (18) quercetin; (19) 3,4-dimethoxybenzoic acid; (20) naringenin; (21) kaempferol.

In this study, the phenolic acids such as phloroglucinol, gallic acid, protocatechuic acid, chlorogenic acid, 2,4-dihydroxybenzoic acid, vanillic acid, syringic acid, *p*-coumaric acid, ferulic acid, salicylic acid, benzoic acid, *o*-coumaric acid, and 3,4-dimethoxybenzoic acid, and flavonoids such as catechin, naringin, hesperidin, myricetin, quercetin, naringenin, kaempferol and rutin were detected in the free and bound phenolic extracts of blue highland barley, using a high-performance liquid chromatography (HPLC) system (Figure 1, Table 3). All of the monomer phenolic substances detected had a large amplitude of variation in content and high variable coefficient, which shows that there was a significant difference among the varieties. Gallic acid, benzoic acid, syringic acid and *p*-coumaric are the main phenolic acids of blue highland barley, their combined sum accounting for 87.56% of the total phenolic acids, which was similar to the value shown in a previous report, in which syringic acid and *p*-coumaric acid were determined to be the main phenolic acids of barley [16,17,32]. However, unlike other current reports, the present study showed that gallic acid and benzoic acid were the main free phenolic acids in blue grain highland barley. Phloroglucinol, 2,4-dihydroxybenzoic acid, vanillic acid, ferulic acid, salicylic acid and *o*-coumaric acid have lower relative average contents in the blue highland barley tested; this is inconsistent with the report results of Abdel-Aal [12] and Zielinski [33], which showed that ferulic acid is the main phenolic acid in barley, wheat and rye. In all of the phenolic acids detected, the contents of protocatechuic acid, chlorogenic acid, 2,4-dihydroxybenzoic acid and vanillic acid in the free phenolic extract of the blue highland barley were respectively 19.0, 2.62, 1.21 and 1.39 times those in the bound phenolic extract, which means that these four types of phenolic acids mainly exist in the free phenolic content of blue highland barley. This is inconsistent with the report results which showed that protocatechuic acid, chlorogenic acid, 2,4-dihydroxybenzoic acid and vanillic acid mainly exist in a bound state in cereals [5]. For the remaining phenolic acids tested, their contents were significantly higher in the bound extracts than in the free ones. Among them, the contents of gallic acid, syringic acid, *p*-coumaric, benzoic acid and 3,4-dimethoxybenzoic acid in the bound extracts were 13.81–102.80 times those of the free content, and these are the main contributors to the content of the bound total phenolic acids.

*o*-Coumaric acid was not detected in the free phenolics extracts in any of the blue highland barley tested, and the detected content of salicylic acid was (1.01–20.55 μg/g DW), while the remaining phenolic acid contents were not detected in the free phenolic extracts of some of the highland barley varieties tested. The following items were detected in the bound phenolic extracts of all of the highland barley varieties tested: *p*-coumaric (14.61–583.54 μg/g DW), ferulic acid (5.61–13.88 μg/g), salicylic acid (7.41–28.38 μg/g DW), benzoic acid (8.81–528.56 μg/g DW), *o*-coumaric acid (15.11–60.26 μg/g DW) and 3,4-dimethoxybenzoic acid (18.51–110.85 μg/g DW). The contents of the remaining phenolic acids were not detected in the bound phenolic extracts of a portion of the highland barley varieties tested. This means that the abovementioned monomer phenolic acids were the prevalent characteristic phenolic acids in the blue highland barley. In addition, there were differences in the phenolic acid contents of the tested main monomers among the different varieties of blue grain highland barley. Among them, the content of gallic acid was rich in Walan (1366.69 ± 2.34 μg/g DW, Beiqing 8 (1173.02 ± 1.32 μg/g DW), Dulihuang (1031.00 ± 1.57μg/g DW) and Beiqing 9 (459.84 ± 1.49 μg/g DW). Chlorogenic acid only had a high content in Ganqing 4 (98.36 ± 1.68 μg/g DW). The existing research has proven that chlorogenic acid was a kind of plant allelochemical found in plants, which could inhibit the growth of weeds and did less harm to soil [34]. Therefore, Ganqing 4 highland barley had the potential to both protect the environment and develop botanical pesticides. The content of syringic acid was rich in Xunhualianglan (916.20 ± 6.02 μg/g DW), Zangqing 320 (657.04 ± 1.24 μg/g DW), Walan (591.99 ± 0.98 μg/g DW) and Beiqing 2 (573.71 ± 1.45 μg/g DW). The *p*-coumaric acid was rich in Ganqing 4 (583.54 ± μg/g DW), Walan (200.34 ± 0.98 μg/g DW), Dulihuang (133.94 ± 4.22 μg/g DW), Zangqing 320 (129.00 ± 1.04 μg/g DW) and Beiqing 9 (100.48 ± 2.05 μg/g DW). The benzoic acid was rich in Beiqing 2 (521.74 ± 1.82 μg/g DW), Xunhualianglan (442.88 ± 2.02 μg/g DW) and Dulihuang (432.17 ± 0.98 μg/g DW). The Zangqing 320, Zangqing 690 and Mennong 1 varieties contained 109.27, 106.16 and 112.99 μg/g of 3,4-dimethoxybenzoic acid, respectively.

The main monomer phenolic acids detected in Beiqing 4 were fewer in number. It was thus indicated that genotype had a significant effect on the composition and content of phenolic acids. The contents of catechin, naringin, hesperidin, myricetin, quercetin, naringenin, kaempferol and rutin were detected in the free and bound extracts of the blue highland barley tested. Among these, naringenin had the highest average content (137.09 ± 172.60 μg/g DW), which is measured for the first time in existing highland barley. This was followed by hesperidin (104.07 ± 81.23 μg/g DW), then by rutin, catechin, quercetin and kaempferol (56.81–69.18 μg/g DW), while naringin (16.71 ± 17.09 μg/g DW) and (15.05 ± 21.81 μg/g DW) had the lowest average contents. This means that naringenin and hesperidin are the main flavonoids in blue highland barley, which is inconsistent with the report results which showed that myricetin is the main flavonoid in colored barley, and naringin and hesperidin have extremely low contents in colored barley [10]. This difference is related to the gene type, planting environment and extraction method of the tested varieties. Among them, genotype had great influence on the content of flavonoids in different blue highland barley. In this study, four kinds of flavonoids of Menyuanlianglan were found in the blue highland barley, including catechin (101.33 ± 1.04 μg/g DW), naringenin (502.48 ± 0.28 μg/g DW), kaempferol (192.27 ± 0.86 μg/g DW) and rutin (141.44 ± 0.56 μg/g DW). The kaempferol (76.89 ± 0.08 μg/g DW) and rutin (118.86 ± 0.10 μg/g DW) were rich in Zangqing 690, catechin (95.44 ± 0.52 μg/g DW) and hesperidin (187.40 ± 0.06 μg/g DW) were rich in Mennong 1, catechin (96.87 ± 0.10 μg/g DW) and quercetin (87.30 ± 0.12 μg/g DW) were rich in Xunhualianglan. Rutin (92.09 ± 0.04 μg/g DW), quercetin (60.41 ± 0.05 μg/g DW), catechin (105.38 ± 0.28 μg/g DW), naringenin (507.70 ± 0.36 μg/g DW) and hesperidin (152.63 ± 0.21 μg/g DW) were respectively rich in Beiqing 2, Beiqing 4, Beiqing 8, Ganqing 4 and Beiqing 9. The content of the tested main flavonoid monomer in Zangqing 320 was low in comparison. Myricetin and quercetin were not detected in the free extracts of the tested blue-grained highland barley. The average content of catechinic acid in the free-state extracts was slightly higher than that in the bound state, but the difference was not significant. However, the average contents of naringin, hesperidin, myricetin, quercetin, naringenin, kaempferol and rutin in the bound extract were about 5.21–51.02 times those of the corresponding free extracts, which means that they mainly exist in the blue highland barley in a bound state. These results are inconsistent with the existence form of flavonoid measured by using the chemical method. A possible reason for this is that the chemical method uses catechin as the standard item to calculate the content of flavonoid in extract, while the measurement using the HPLC system result shows that the content of catechin in the free extracts is slightly higher than in the bound content, which leads to the result that the content of flavonoid in the free phenolic extract measured by using the chemical method is higher than the bound content.

Table 4 compares the free and combined individual phenolic compounds of the blue highland barley planted in different areas. It is shown that for the blue highland barley planted in different locations, the contents of free, bound and total phenolic compounds have significant differences (*p* < 0.05). There are 11 kinds of free phenolic acids and 12 kinds of bound phenolic acid which were detected from the blue highland barley planted in Menyuan (Qinghai) and Hezuo (Gansu), and eight kinds of free phenolic acids and nine kinds of bound phenolic acid detected from the blue highland barley planted in Lhasa (Tibet). The average contents of free, bound and total phenolic acids are the highest (201.54 ± 8.94, 1498.69 ± 102.58, 1623.69 ± 100.91 ug/g DW) in the blue highland barley planted in Menyuan (Qinghai), followed by those in Hezuo (Gansu) (102.47 ± 9.35, 1289.05 ± 228.76, 1392.52 ± 227.57 ug/g DW), with the lowest those planted in Lhasa (Tibet) (87.79 ± 10.25, 914.00 ± 125.77, 1001.79 ± 124.80 ug/g DW). For the blue highland barley planted in Menyuan (Qinghai), Lhasa (Tibet) and Hezuo (Gansu), respectively six, four and four kinds of free flavonoids detected, and eights kinds of flavonoid monomers were detected from the bound extract of the blue highland barley planted in the three locations. For the free flavonoids, the content of the blue highland barley planted in Menyuan (Qinghai) is the highest (76.54 ± 15.24 ug/g DW), while the content of bound and total flavonoids of the blue highland barley planted in Hezuo (Gansu) is the highest (559.78 ± 66.52 ug/g DW, 594.24 ± 69.70 ug/g DW). These results are consistent with the contents of free, bound and total phenolics detected by chemical methods. Therefore, it is indicated that the blue highland barley planted in Menyuan (Qinghai) includes more abundant types of phenolic compounds and higher content of phenolic acids, while that planted in Hezuo (Gansu) includes a higher content of flavonoids, which provides a reliable basis for the selection of the materials for different purposes. The contents of gallic acid, protocatechuic acid, 2,4-dihydroxybenzoic acid and catechin in the blue highland barley planted in Menyuan (Qinghai) are relatively high, those of phloroglucinol, chlorogenic acid, *p*-coumaric, naringin, hesperidin and myricetin in that planted in Hezuo (Gansu) are rich, and the contents of syringic acid, benzoic acid and rutin in that planted in Lhasa (Tibet) are rich.

Therefore, it can be indicated that the planting environment exhibits a significant impact on the monomer composition and content of polyphenol in blue highland barley. Consequently, we must give full consideration to environmental factors while selecting the raw materials of blue highland barley according to specific needs.

### 2.3. Antioxidant Properties

**Figure 2:**
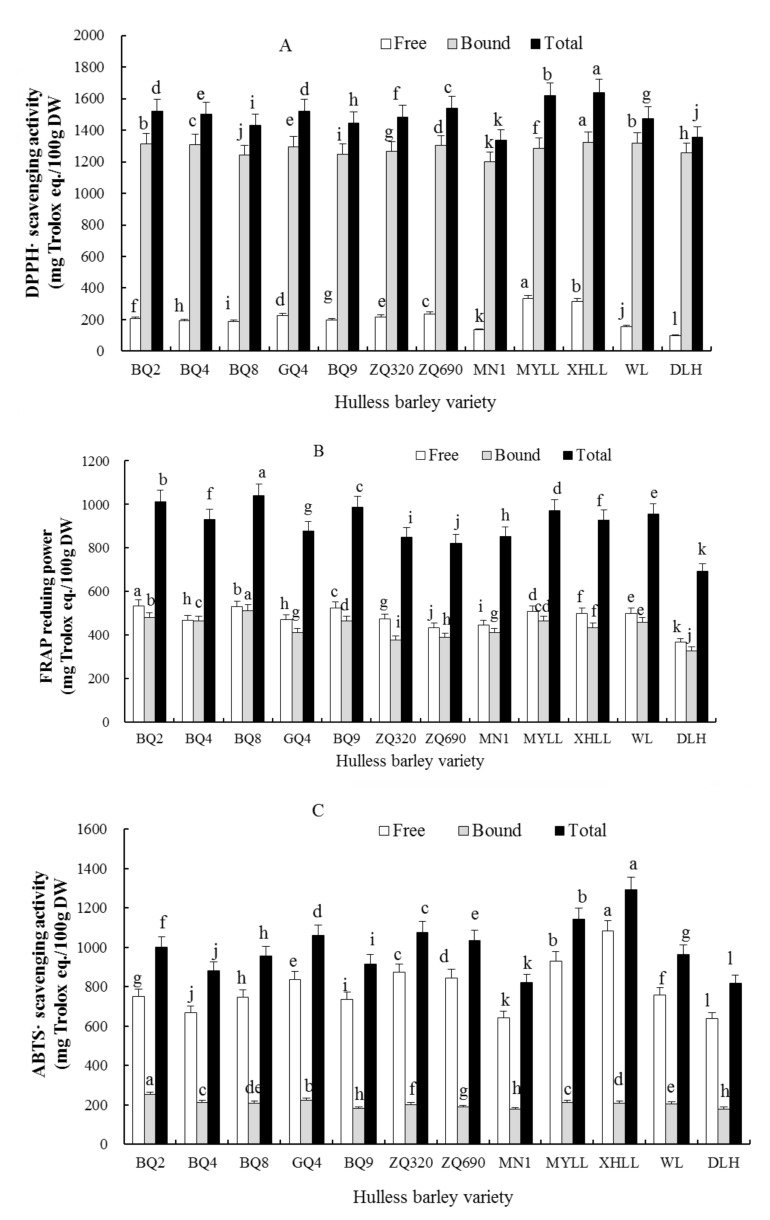
Analysis of the antioxidant activity of different varieties of blue highland barley. (**A**) DPPH radical scavenging activity (**B**) FRAP reduing power (**C**) ABTS^•+^ scavenging activity. Different letters denote significant difference at the level *p* < 0.05. BQ2, Beiqing 2; BQ4, Beiqing 4; BQ8, Beiqing 8; GQ4, Ganqing 4; BQ9, Beiqing 9; ZQ320, Zangqing 320; ZQ690, Zangqing 690; MN1, Mennong 1; MYLL, Menyuanlianglan; XHLL, Xunhualianglan; WL, Walan; DLH, Dulihuang.

**Figure 3:**
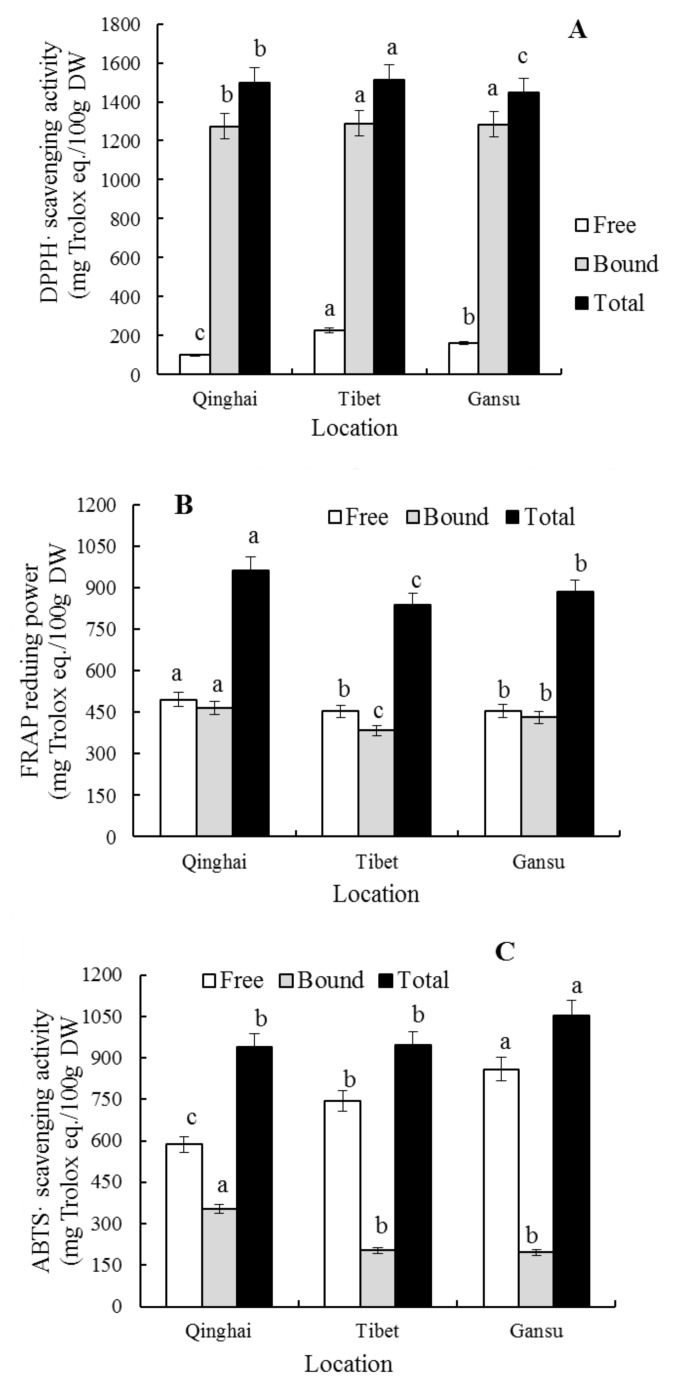
Analysis of the antioxidant activity of blue highland barley from different locations. (**A**) DPPH radical scavenging activity; (**B**) FRAP reducing power; (**C**) ABTS^•+^ scavenging activity. Different letters denote significant difference at the level *p* < 0.05.

Among the three antioxidant systems, the polyphenols in blue highland barley show a relatively higher capacity to scavenge DPPH^•^ (1336.51–1640.01 mg/100 g DW) and ABTS^•+^ (817.41–1291.78 mg/100 g DW). It also has a relatively stronger ferric reducing antioxidant power (FRAP) (693.41–1041.02 mg/100 g DW) (Figure 1, Figure 2 and Figure 3), which is significantly higher than the antioxidant activity of Canadian, Egyptian [35] and Tunisian [14] barley of the same unit, as well as rice [36] and carrot [37] with different colors. Therefore, blue highland barley can be used as a potential and good dietary source for scavenging free radicals.

According to Figure 2, the capacity of bound phenolic extracts in blue highland barley to scavenge the DPPH^•^ radical is much stronger than the free phenolic extracts, but the free phenolic extract has a higher total DPPH^•^ radical scavenging capacity and FRAP than the bound phenolic extract. Among these, the average contribution of the bound phenolic extract to the total DPPH^•^ radical scavenging capacity is greater than 86%, and the average contribution of free phenolic extract to total ABTS^•+^ radical scavenging capacity is greater than 79%. Therefore, it can be concluded that the bound phenolic extract of blue highland barley is the main contributor to the scavenging of DPPH^•^ radical, and free phenolic extract is the main contributor to the scavenging of ABTS^•+^ radical. Free phenolic extract (53%) and bound phenolic extract (47%) have equivalent contribution to the iron ion reducing capacity. In addition, different varieties of blue highland barley have significant differences in the scavenging capacity of DPPH^•^ and ABTS^•+^ and FRAP capacity (*p* < 0.05). Among them, Xunhualianglan has the highest value of scavenging the DPPH^•^ and ABTS^•+^ radical, and Beiqing 8 has the highest total FRAP. This kind of difference is related to the composition and content of different forms of phenolic substances in different varieties of highland barley.

The capabilities of the free and bound polyphenol extraction of blue highland barley planted in the different areas in removing DPPH^•^ (1336.51–1640.01 mg/100 g) and ABTS^•+^ (817.41–1291.78 mg/100 g) and reducing FRAP iron ion are significantly different (*p* < 0.05) (Figure 3). The free (227.24 ± 29.70 mg/100 g) and bound phenolic (1288.67 ± 62.20 mg/100 g) of the blue highland barley planted in Lhasa (Tibet) have a strong ability to remove DPPH^•^, while the free (495.10 ± 34.28 mg/100 g) and bound phenolics (465.84 ± 28.56 mg/100 g) of that planted in Menyuan (Qinghai) have a strong ability to reduce FRAP iron ion, and the bound phenolics (353.75 ± 42.35 mg/100 g) of that planted in Menyuan (Qinghai) and the free phenolics (859.21 ± 61.54 mg/100 g) of that planted in Hezuo (Gansu) have a strong ability to remove ABTS^•+^. Polyphenols are the main contributors to the antioxidant activity of blue highland barley, and the different planting environment affects the compositions and contents of different polyphenols of blue highland barley, and leads to differences in the antioxidant activity. Therefore, it can be concluded that planting environment plays an important role in the strength of the antioxidant activity of highland barley, which is consistent with the results reported by Abdel-Aal, E.M. [15].

### 2.4. Correlations between Phenolic Compounds and Antioxidant Activities

In order to clarify the relationship between the contents of the free and bound phenolic compounds and their antioxidant activities in blue highland barley, correlation analysis was carried out, and the analysis results are shown in Table 5 and Table 6. The free phenolic content showed an extremely significant positive correlation with the DPPH^•^ and ABTS^•+^ radical scavenging capacity and the FRAP *(p* < 0.01). The content of free total phenolic compounds showed a significant positive correlation with the FRAP capacity. The hydroxybenzoic acid and protocatechuic acid in the mon*o*-phenolic content of free extracts show an extremely significant (*p* < 0.01 and *p* < 0.05, respectively) positive correlation with the ABTS^•+^ radical scavenging capacity, and the hydroxybenzoic acid shows a significant positive correlation with the DPPH^•^ radical scavenging capacity. This signifies that hydroxybenzoic acid and protocatechuic acid are the main contributors to the DPPH^•^ and ABTS^•+^ radical scavenging capacity. However, the content of gallic acid shows a significant negative correlation with the ABTS^•+^ radical scavenging capacity, which is consistent with the research results of Zhao [21], which showed that naringenin had a significant negative correlation with the FRAP capacity.

The content of bound phenolics has an extremely significant positive correlation with the ABTS^•+^ radical scavenging capacity and FRAP capacity, while the bound flavonoid content has a significant positive correlation with the ABTS^•+^ radical scavenging capacity. This means that the bound phenolic extract of the blue highland barley contains a greater number of phenolic substances scavenging the ABTS^•+^ radical. Chlorogenic acid, vanillic acid, ferulic acid and quercetin have a significant positive correlation with the ABTS^•+^ radical scavenging capacity, which signifies that they are the main contributors to the ABTS^•+^ radical scavenging capacity. This is similar to the results of Zhao, H.F. [21], which showed that ferulic acid and vanillic acid were the main contributors to the ABTS^•+^ radical scavenging capacity of the barley extract. In addition, we also observed that ferulic acid had a significant positive correlation with the FRAP capacity. This means that ferulic acid has a very strong antioxidant activity, despite the fact that its content in the blue highland barley is not high, and it is the main antioxidant substance in the bound phenolic extract of blue highland barley. This is consistent with the research results of Abdel-Aal, EM [12]. Quercetin had a significant positive correlation with the DPPH^•^ radical scavenging capacity, which signifies that quercetin is the main contributor to the DPPH^•^ radical scavenging capacity of bound phenolic extract of the blue highland barley. 3,4-Dimethoxybenzoic acid had a significant negative correlation with the FRAP capacity, and hesperidin had a significant negative correlation with the ABTS^•+^ free radical scavenging capacity. This signifies that different kinds of monomer phenolics exhibit selectivity for different antioxidant activity evaluation methods.

In addition, we observed that there was an extremely significant positive correlation between the free phenolic content and free flavonoid content under the test condition (*p* < 0.01), which means that the higher the content of free phenolic in the blue highland barley was, the higher the contained content of free flavonoid would be. The content of catechin had a significant positive correlation with the content of free phenolic, naringenin had a significant negative correlation with the content of free phenolic, and the contents of ferulic acid, quercetin and rutin had significant positive correlations with the bound phenolic. Therefore, we can evaluate the contents of catechin and naringenin in the blue highland barley according to the content of the free phenolic, and evaluate the content of ferulic acid, quercetin and rutin according to the content of the bound phenol. All of these provide a simple method by which to select the special variety of highland barley. In addition, the content of protocatechuic acid had a significant positive correlation with the content of free type total phenolic compounds, which signifies that the higher the content of protocatechuic acid in the blue highland barley was, the higher the content of its free total phenolic acid would be. In accordance with the positive correlation of the content of phloroglucinol, pcoumaric acid and myricetin with the content of the bound total phenolic acid, the higher the contents of phloroglucinol, *p*-coumaric acid and myricetin were, the higher the content of bound total phenolic acid of the blue highland barley would be. However, in future research, a larger sample number must be used to investigate or further verify the correlation between monomeric phenolics and total phenolic acids in blue highland barley.

## 3. Materials and Methods

### 3.1. Highland Barley Sample Preparation

The 12 highland barley varieties (Beiqing 2, Beiqing 4, Beiqing 8, Ganqing 4, Beiqing 9, Zangqing 320, Zangqing 690, Mennong 1, Menyuanlianglan, Xunhualianglan, Walan and Dulihuang) were grown at three different locations. The Beiqing 2, Beiqing 4, Beiqing 8, Mennong 1, Menyuanlianglan, Xunhualianglan and Dulihuang were cultivated by the Haibei Institute of Agricultural Science in Qinghai; the Ganqing 4 and Walan were cultivated by the Ganzi Institute of Agricultural Science in Gansu; and the Zangqing 320 and Zangqing 690 were cultivated by the Tibet Academy of Agricultural and Animal Husbandry Sciences in Tibet. They were planted at Menyuan in Qinghai (mean temperature: 10.50 °C, altitude: 2700 m), Hezuo in Gansu (mean temperature: 15.20 °C, altitude: 2960 m), Lhasa in Tibet (mean temperature: 18.60 °C, altitude: 3658 m), on 14 April, 18 March and 25 March 2016, respectively. All of the blue highland barley samples were collected by one of the authors (Associate Prof. B. Dang). These materials were identified by Researcher Kulun Wu, Qinghai Academy of Agricultural and Forestry Sciences, and were deposited in the National Duplicate Genebank for Crops, Qinghai Academy of Agricultural and Forestry Sciences. All samples number is QHZQK-00111-00111. All samples were dried at room temperature and ground using an FW100-High Speed Universal Grinder (Huaxin, Tianjin, China) until they passed through a 40-mesh sieve, then stored at −20 °C until use.

### 3.2. Chemicals

1,1-Diphenyl-2-picrylhydrazylradical (DPPH^•^), 2,4,6-tripyridyl-s-triazine (TPTZ), 2,2′-azinobis-(3-ethylbenzthiazoline-6-sulfonate) (ABTS), and 6-hydroxy-2,5,7,8-tetramethylchroman-2-carboxylic acid (Trolox) were purchased from Sigma (St. Louis, MO, USA). Phloroglucinol, gallic acid, protocatechuic acid, chlorogenic acid, 2,4-dihydroxybenzoic acid, vanillic, syringic acid, *p*-coumaric, ferulic acid, salicylic acid, benzoic acid, *o*-coumaric acid, 3,4-dimethoxybenzoic acid, catechin, naringin, hesperidin, myricetin, quercetin, naringenin, kaempferol and rutin were purchased from Shanghai Yuanye Bio-Technique Co. Ltd. (Shanghai, China). The Folin-Ciocalteu reagent was purchased from Beijing Solarbio Science & Technology Co. Ltd. (Beijing, China). All other chemicals and solvents were of analytical grade.

### 3.3. Extraction of Free and Bound Phenolic Compounds from Highland Barley

The free and bound phenolic compounds were extracted by a previously reported method [21,38] with some improvements. The whole powder of highland barley (1.0 g) was extracted with 80% aqueous acetone (25 mL) for 20 min using an ultrasonic cleaner (KQ-500DE, Kunshan, Jiangsu, China) at room temperature. The mixture underwent refrigerated centrifugation at 3000 g for 10 min using a 5430R centrifuge (Eppendorf, Hamburg, Germany), then the supernatant was collected. The extraction was repeated three times. The supernatants were merged, and vacuum evaporated to dryness at 45 °C, and finally reconstituted with methanol to a volume of 10 mL. The extracts were stored by avoiding light at −20 °C until use. 

We added 20 mL normal hexane to the residues after extraction of free phenolic compounds. After centrifugation at 1500× *g* for 5 min to separate the supernatant, the residues were treated with 17 mL of methanol/H_2_SO_4_ (90:10, *v/v*) at 70 °C for 1 h. The final solution was extracted five times with ethyl acetate (20 mL). The organic fractions were combined and evaporated to dryness in vacuo at 45 °C, before being reconstituted with methanol and stored at −20 °C until use.

### 3.4. Determination of Total Phenolic Content

The total phenolic contents were determined by means of the Folin-Ciocalteu (FC) colorimetric method previously described by Adom et al., with minor modifications [39]. In brief, the extracts or control (125 μL) were mixed with distilled deionized water (500 μL), then FC reagent (125 μL) was added. After 6 min, 1.25 mL of a 7% Na_2_CO_3_ solution was added to the mixture, followed by 1.0 mL of water, to bring the final volume to 3.0 mL. After 90 min of incubation at room temperature in the dark, the absorbance at 760 nm was measured using a spectrometer (UV1240, Shimadzu, Kyoto, Japan). Gallic acid was used as the standard, and the total phenolic contents were expressed as mg gallic acid equivalents (GAE)/100 g DW.

### 3.5. Determination of Total Flavonoid Content

The total flavonoid contents were determined via a colorimetric method previously described, with some modifications made in our laboratory [39]. In brief, an aliquot (100 μL) of each extract or a standard solution was mixed with 5% NaNO_2_ solution (200 μL). After 6 min, a 10% AlCl_3_·6H_2_O solution (200 μL) was added to each mixture. After 6 min, a 1 M NaOH solution (2.0 mL) was added, and the total volume was adjusted to 5.0 mL with deionised water. (+)-Catechin was used as a standard. The absorbance at 510 nm, which was corrected using a blank, was then determined, and the results were expressed as mg of (+)-catechin equivalents (CE)/100 g DW.

### 3.6. Analysis of Phenolic Compound Compositions in Blue Highland Barley

The 12 blue highland barley samples were analyzed for their free, bound and total (free and bound) phenolic acid and flavonoid composition by means of HPLC (Waters 600E, Delta 600 pump, Waters, Milford, CT, USA), using a Phenomenex C18 column (250 mm × 4.6 mm) and UV/VIS detector [10]. Each injection was monitored at 280 nm, the run time was 60 min, and the flow rate was maintained at 0.8 mL/min using an injection volume of 20 μL. The mobile phase was distilled water with 0.1% glacial acetic acid (solvent A) and acetonitrile with 0.1% glacial acetic acid (solvent B). The following gradient was used: 0 min, 8% B in A; 2 min, 10% B in A; 27 min, 30% B in A; 50 min, 90% B in A; 51–56 min, 100% B in A; and 51–60 min, 8% B in A. The identity of each peak was confirmed based on the retention time determined for each pure compound. The values are expressed as μg/g DW.

### 3.7. Determination of Antioxidant Activities

#### 3.7.1. DPPH Radical Scavenging Activity Assay

The DPPH radical scavenging activity of the blue highland barley phenolics were determined using the colorimetric method described by Abu et al., with some modifications [40]. We placed 1 mL of the sample extract solution in a test tube, then added 4.5 mL 0.1 mmol/L of DPPH radical solution; caused it to react 30 min in the dark after thorough mixing, using methyl alcohol for the blank zero setting instead of a sample extraction solution; measured the absorbance under the wave length of 517 nm; and repeated this process 3 times. Then, a standard curve was prepared by plotting the percentage of the free radical scavenging activity of Trolox versus its concentration (1–140 μmol/L). The DPPH radical scavenging activity of the sample was expressed as μmol Trolox equivalent to the antioxidant capacity in 100 g DW of the sample (μmol Trolox eq./100 g DW).

#### 3.7.2. Ferric Reducing Antioxidant Power Assay

The ferric reducing antioxidant power assays were determined using the method described by Benzie et al. with some modifications [41]. The preparation of ferric reducing antioxidant power assay (FRAP) working solution was as follows: 300 mmol·L^−1^ pH 3.6 sodium acetate buffer solution (3.0762 g C_2_H_3_NaO_2_· plus 20 mL C_2_H_4_O_2_, which was adjusted to the volume of 250 mL with distilled water), 10 mmol·L^−1^ TPTZ solution (0.1562 g TPTZ, volume fixed to 100 mL with 40 mmol·L^−1^ hydrochloric acid), and 20 mmol·L^−1^ FeCl_3_ solution. These three solutions were then mixed according to the volume ratio of 10:1:1, and preheated in a 37 °C water bath kettle for standby before application.

Each sample extraction solution or control (50 μL) was mixed with the FRAP working solution (4.5 mL). Absorbance was measured at 593 nm after 30 min of incubation at room temperature in the dark. A standard curve was then prepared by plotting the percentage of the free radical scavenging activity of Trolox versus its concentration (1–300 μmol/L). The FRAP of the sample was expressed as μmol Trolox eq./100 g DW.

#### 3.7.3. ABTS^•+^ Free Radical Scavenging Capacity

The free radical scavenging capacity of the extracts was evaluated against ABTS^•+^ generated according to method described by Guo et al. [27]. The preparation of the ABTS^•+^ working solution was as follows: We mixed the 5 mL 7 mmol/L solution with 88 μL 140 mmol/L potassium persulfate solution, and let the mixed solution stand for 11–16 h without light and at room temperature, in order to obtain the ABTS^+•^ stock solution. We then mixed this stock solution with absolute methanol according to a suitable proportion (1:100 (*v/v*)), and its light absorption value at A_734 nm_ was required to reach 0.7 ± 0.02, so as to obtain the ABTS^+•^ working solution for standby before application.

Each sample extraction solution or control (200 μL) was mixed with the ABTS^•+^ working solution (4 mL). Absorbance was measured at 734 nm after 30 min of incubation at room temperature in the dark. A standard curve was then prepared by plotting the percentage of the free radical scavenging activity of Trolox versus its concentration (1–300 μmol/L). The ABTS^•+^ scavenging activity of the sample was expressed as μmol Trolox eq./100 g DW.

### 3.8. Statistical Analysis

All samples were measured and analyzed in triplicate. The data were reported as mean ± SD (standard deviation). The results of the mean, range and variable coefficient (CV) of the phenolic and flavonoid contents was analyzed using Excel 2003 (Microsoft, Redmond, WA, USA). The analyses of variance and significance differences among the means were analyzed by an SNK-q test, the correlation coefficients of the data were analyzed using a Pearson test, and statistical analyses were performed using SPSS statistical package version 21.0 (SPSS Inc., Chicago, IL, USA). Statistical significance was defined as *p* < 0.05.

## 4. Conclusions

The results of this study show that the blue highland barley grains are rich in phenolic compounds and have a strong antioxidant activity. In addition, there were significant differences among the different varieties, which signifies that the gene type of blue highland barley is the main factor influencing the content of free and bound phenolic in the grains.

In the blue highland barley grains, the percentage of free phenolic in the total phenolic is equivalent to the bound phenolic, which signifies that the phenolic substances of the blue highland barley coexist in both free and bound forms. This result will aid in understanding the health benefits of consumption of blue highland barley. The phenolic compounds in the blue highland barley seeds mainly consist of gallic acid, benzoic acid, syringic acid, *p*-coumaric acid, naringenin, hesperidin, rutin, catechin and quercetin. Among these, the free phenolics mainly consist of protocatechuic acid, chlorogenic acid and catechin, while the bound phenolics mainly consist of gallic, syringic acid, *p*-coumaric acid, benzoic acid, 3,4-dimethoxybenzoic acid, naringenin, hesperidin, quercetin and rutin. In addition, planting environment exerts a very important influence on the composition, content and antioxidant activity of the free and combined polyphenol of blue highland barley.

The correlation analysis results show that the hydroxybenzoic acid and protocatechuic acid in the free phenolic extracts are the main contributors to the DPPH^•^ and ABTS^•+^ radical scavenging capacity, while chlorogenic acid, vanillic acid, ferulic acid and quercetin in the bound phenolic extracts are the main contributors to the free radical scavenging capacity. These results reveal the fact that blue highland barley can be used as a source of natural antioxidant food, and can also be used to guide us to increasing the development and daily consumption of foods related to blue highland barley.

## Figures and Tables

**Table 1:** Phenolic and flavonoid contents of blue Highland barley.

Variety	Phenolic Content (mg/100 g DW)			Flavonoid Content (mg/100 g DW)		
	Free	Bound	Total	Free	Bound	Total
Beiqing 2	213.19 ± 11.86 abB	240.75 ± 5.44 aA	453.94 ± 11.38 a	25.59 ± 1.63 bA	22.38 ± 0.37 aB	47.98 ± 2.00 a
Beiqing 4	190.15 ± 13.23 bcB	227.25 ± 3.97 abA	417.40 ± 10.58 de	20.63 ± 0.94 eA	19.72 ± 0.94 bcA	40.35 ± 0.00 bc
Beiqing 8	225.73 ± 11.98 abA	223.47 ± 8.98 abA	449.20 ± 2.99 ab	25.29 ± 0.93 bA	18.41 ± 1.36 cdeB	43.70 ± 2.29 ab
Ganqing 4	209.09 ± 9.12 abcB	216.41 ± 1.17 bcA	425.51 ± 5.72 cd	24.27 ± 0.01 bcA	18.80 ± 1.11 cdeB	43.07 ± 1.11 abc
Beiqing 9	215.25 ± 19.24 abA	187.90 ± 3.89 deB	403.14 ± 5.73 ef	24.00 ± 2.37 bcA	17.39 ± 0.59 deB	41.39 ± 1.78 bc
Zangqing 320	207.46 ± 3.09 abcA	177.11 ± 0.00 eB	384.57 ± 1.54 g	22.93 ± 0.22 cdA	16.78 ± 1.10 efB	39.71 ± 0.88 bc
Zangqing 690	219.24 ± 8.57 abA	185.65 ± 7.59 deB	404.88 ± 3.31 f	28.11 ± 0.82 aA	14.95 ± 0.67 fB	43.07 ± 1.49 abc
Mennong 1	186.75 ± 6.97 bcB	213.87 ± 2.20 bcA	400.62 ± 5.69 f	21.18 ± 1.26 deA	16.76 ± 0.49 efB	37.93 ± 0.77 bc
Menyuanlianglan	222.95 ± 13.80 abA	223.62 ± 5.96 abA	446.58 ± 0.94 bc	22.68 ± 0.07 cdeA	18.59 ± 1.39 cdeB	41.27 ± 1.47 bc
Xunhualianglan	237.60 ± 0.20 aA	199.93 ± 5.73 cdB	437.53 ± 5.63 c	24.67 ± 0.38 bcA	19.34 ± 0.08 cdB	44.02 ± 0.45 ab
Walan	197.49 ± 11.90 abcB	219.26 ± 0.19 bA	416.75 ± 5.76 de	22.96 ± 0.31 cdA	18.66 ± 0.81 cdeB	41.61 ± 0.50 bc
Dulihuang	166.20 ± 10.46 cB	170.10 ± 0.19 eA	336.29 ± 5.42 h	21.58 ± 0.15 deA	20.96 ± 1.60 abA	42.49 ± 1.46 bc
Mean	207.59 ± 19.65	207.11 ± 22.38	414.70 ± 32.86	23.60 ± 2.18	18.61 ± 2.06	42.22 ± 2.53
Range	166.20–237.60	170.10–240.75	336.29–453.94	20.61–28.11	14.91–22.38	37.93–47.98
CV%	9.5	10.8	7.9	9.3	11.1	6.0

The data in the table are the average value of three repetitions; the lowercase letters designate significant differences among different varieties (*p* < 0.05), and the capital letters show significant differences between the free and bound substances of the same variety (*p* < 0.05).

**Table 2:** Average contents of phenolic and flavonoid of blue highland barley from different locations.

	Location	Free	Bound	Total
		Mean	Mean	Mean
Phenolic content (mg/100 g DW)	Qinghai	213.09 ± 18.63 a	216.68 ± 17.80 a	429.77 ± 22.42 a
	Tibet	213.35 ± 8.33 a	181.38 ± 6.04 c	394.73 ± 14.36 b
	Gansu	190.93 ± 22.19 b	201.92 ± 27.60 b	392.85 ± 49.18 c
Flavonoid content (mg/100 g DW)	Qinghai	23.43 ± 1.98 b	18.94 ± 1.83 a	42.38 ± 3.21 a
	Tibet	25.52 ± 3.66 a	15.87 ± 1.29 b	41.39 ± 2.38 b
	Gansu	22.71 ± 1.69 b	19.68 ± 1.65 a	42.39 ± 0.74 a

The data in the table are the average values of three repetitions; the lowercase letters designate significant differences among different locations (*p* < 0.05).

**Table 3:** Composition and content of phenolic acid and flavonoids in blue highland barley grains.

	Free (ug/g DW)			Bound (ug/g DW)			Total (ug/g DW)		
	Mean	Range	CV/%	Mean	Range	CV/%	Mean	Range	CV/%
Phenolic Acids									
Phl ^a^	3.71 ± 10.03 b ^b^	1–34.11	270.32	8.04 ± 9.58 a	1–27.18	119.2	11.75 ± 17.20	1–61.29	146.44
Gal	9.11 ± 13.49 b	1–29.71	148.1	338.29 ± 528.58 a	1–1366.09	156.25	347.40 ± 529.38	1–1366.69	152.38
Dih	24.98 ± 45.39 a	1–153.99	181.67	1.31 ± 4.53 b	1–15.70 b	346.41	26.29 ± 44.83	1–153.99	170.49
Chl	28.32 ± 16.22 a	1–46.61	57.27	10.82 ± 18.89 b	1–61.55	175.03	39.13 ± 23.86	1–98.36	60.98
Res	6.53 ± 11.45 a	1–37.08	175.32	5.39 ± 6.73 a	1–15.60	124.88	11.92 ± 15.65	1–49.37	131.37
Van	5.10 ± 6.86 a	1–25.02	134.48	3.66 ± 13.56 b	1–19.09	371.03	8.75 ± 13.56	1–39.14	154.94
Syr	3.44 ± 5.23 b	1–13.20	151.85	267.48 ± 329.25 a	1–916.20	123.09	270.93 ± 328.22	1–916.20	121.14
Pco	9.25 ± 12.81 b	1–43.36	138.43	127.92 ± 155.06 a	14.61–583.54	121.22	137.17 ± 152.54	15.71–583.54	111.20
Fer	2.52 ± 3.74 b	1–8.11	148.66	8.94 ± 2.82 a	5.61–13.88	31.50	11.46 ± 5.35	5.91–21.99	46.71
Sal	8.34 ± 4.48 b	1.01–20.50	43.13	17.50 ± 8.55 a	7.41–28.38	48.81	25.85 ± 9.91	7.71–35.64	38.35
Ben	2.78 ± 4.12 b	1–9.32	148.27	285.79 ± 235.04 a	8.81–528.56	82.24	288.57 ± 234.52	8.81–528.56	81.27
oCo	nd	nd	nd	23.88 ± 13.75 a	15.11–60.26	57.57	23.88 ± 13.75	15.11–60.26	57.57
Dim	9.00 ± 9.79 b	1–20.77	108.72	66.65 ± 35.08 a	18.51–110.85	52.6 ± 0.88	75.65 ± 35.45	21.1–112.99	46.86
Total	104.08 ± 0.51 b			1165.67 ± 1.22 a			1278.75		
Flavonoids									
Cat	33.09 ± 27.95 a	1–70.24	84.46	31.3 ± 11.14 a	1–49.11	35.60	64.39 ± 32.93	1–105.38	51.14
Nar	2.37 ± 3.56 b	1–7.94	150.18	14.34 ± 15.54 a	1–61.78	108.39	16.71 ± 17.09	7.71–69.72	102.28
Hes	2.01 ± 5.38 b	1–18.22	267.23	102.05 ± 78.96 a	5.01–230.86	77.37	104.07 ± 81.23	5.01–249.08	78.05
Myr	nd	nd	nd	15.05 ± 21.81 a	6.51–83.80	144.93	15.05 ± 21.81	6.51–83.80	144.93
Que	nd	nd	nd	62.56 ± 61.12 a	26.41–87.30	97.70	62.56 ± 61.12	26.41–87.30	97.70
Nari	8.25 ± 3.70 b	4.51–14.90	44.81	128.83 ± 173.45 a	33.71–501.45	134.62	137.09 ± 172.60	47.1–502.48	125.91
Kae	8.60 ± 7.66 b	1–17.79	89.09	48.22 ± 44.19 a	19.61–178.21	91.63	56.82 ± 45.75	19.61–192.27	80.53
Rut	11.01 ± 5.55 b	3.21–20.55	50.40	58.17 ± 41.64 a	11.21–106.97	71.58	69.18 ± 42.12	13.81–117.95	60.89
Total	65.33 ± 0.12 b			460.52 ± 1.75 a			525.87 ± 0.80		

^a^ Phl, phloroglucinol; Gal, gallic acid; Pro, protocatechuic acid; Chl, chlorogenic acid; Dih, 2,4-dihydroxybenzoic acid; Van, vanillic; Syr, syringic acid; pCo, *p*-coumaric; Fer, ferulic acid; Sal, salicylic acid; Ben, benzoic acid; oCo, *o*-coumaric acid; Dim, 3,4-dimethoxybenzoic acid; Cat, catechin; Nar, naringin; Hes, hesperidin; Myr, myricetin; Que, quercetin; Nari, naringenin; Kae, kaempferol; Rut, rutin. ^b^ Means in the same line with different letters are significantly different (*p* < 0.05) between the free and bound content of phenolic acid and flavonoids. nd, not detected.

**Table 4:** Composition and content of phenolic acid and flavonoids in blue highland barley grains from different locations.

		Free (ug/g DW)	Bound (ug/g DW)	Total (ug/g DW)
Phenolic Acids				
Phl ^a^	Qinghai	1.49 ± 0.94 b	5.45 ± 2.90 b	6.94 ± 3.65 b
	Tibet	nd	4.86 ± 1.87 b	4.86 ± 1.87 c
	Gansu	11.37 ± 9.69 a	16.20 ± 14.32 a	27.57 ± 11.10 a
Gal	Qinghai	11.84 ± 4.82 a	790.43 ± 78.07 a	812.27 ± 38.17 a
	Tibet	nd	nd	nd
	Gansu	8.80 ± 5.24 b	241.17 ± 40.16 b	249.97 ± 72.21 b
Dih	Qinghai	30.91 ± 9.04 a	nd	30.91 ± 9.04 a
	Tibet	9.88 ± 3.97 c	nd	9.88 ± 3.97 c
	Gansu	21.23 ± 10.08 b	5.23 ± 2.06 a	26.46 ± 12.33 b
Chl	Qinghai	23.69 ± 18.98 c	6.78 ± 2.59 b	30.47 ± 16.66 c
	Tibet	38.22 ± 17.41 a	nd	38.22 ± 17.41 b
	Gansu	32.51 ± 3.84 b	27.44 ± 11.31 a	59.94 ± 35.14 a
Res	Qinghai	9.31 ± 3.98 a	7.00 ± 6.57 a	16.31 ± 8.79 a
	Tibet	nd	7.82 ± 5.05 a	7.82 ± 5.05 b
	Gansu	4.39 ± 1.60 b	nd	4.39 ± 1.60 c
Van	Qinghai	5.27 ± 2.77 b	4.74 ± 1.23 a	10.01 ± 7.26 a
	Tibet	8.43 ± 1.92 a	nd	8.43 ± 1.92 b
	Gansu	2.48 ± 1.30 c	3.55 ± 1.15 b	6.03 ± 2.46 c
Syr	Qinghai	4.81 ± 2.09 a	236.53 ± 62.62 c	241.33 ± 62.30 c
	Tibet	3.83 ± 1.42 b	328.52 ± 64.60 a	332.35 ± 59.18 a
	Gansu	nd	299.03 ± 92.00 b	299.03 ± 92.00 b
Pco	Qinghai	12.31 ± 5.59 a	71.00 ± 54.95 b	83.30 ± 54.26 b
	Tibet	5.58 ± 1.89 b	66.97 ± 11.94 c	72.55 ± 19.83 c
	Gansu	4.57 ± 2.92 c	301.37 ± 45.78 a	305.94 ± 42.69 a
Fer	Qinghai	3.15 ± 0.97 a	9.89 ± 2.52 a	13.04 ± 6.04 a
	Tibet	nd	6.55 ± 0.91 b	6.55 ± 0.91 c
	Gansu	2.70 ± 1.68 ab	8.34 ± 3.84 ab	11.04 ± 3.83 b
Sal	Qinghai	8.65 ± 5.82 a	20.34 ± 7.96 a	28.84 ± 10.26 a
	Tibet	7.85 ± 0.29 b	7.60 ± 0.21 c	15.45 ± 0.08 c
	Gansu	8.29 ± 0.23 a	17.51 ± 9.25 b	25.79 ± 9.22 b
Ben	Qinghai	2.49 ± 1.26 b	269.02 ± 48.50 c	271.51 ± 50.11 b
	Tibet	3.87 ± 0.47 a	361.83 ± 59.46 a	365.70 ± 53.98 a
	Gansu	2.73 ± 0.72 b	274.21 ± 16.84 b	276.94 ± 12.81 b
oCo	Qinghai	nd	23.96 ± 16.14 b	23.96 ± 16.14 b
	Tibet	nd	32.26 ± 5.12 a	32.26 ± 5.12 a
	Gansu	nd	18.11 ± 2.16 c	18.11 ± 2.16 c
Dim	Qinghai	11.08 ± 10.43 a	53.55 ± 37.22 c	64.63 ± 38.31 c
	Tibet	10.13 ± 4.33 b	97.59 ± 12.13 a	107.72 ± 2.20 a
	Gansu	3.40 ± 0.89 c	76.59 ± 28.82 b	79.99 ± 32.04 b
Total	Qinghai	125.00 ± 8.94 a	1498.69 ± 102.58 b	1623.69 ± 100.91 b
	Tibet	87.79 ± 10.25 c	914.00 ± 125.77 c	1001.79 ± 124.80 c
	Gansu	102.47 ± 9.35 b	1289.05 ± 228.76 a	1392.52 ± 227.57 a
Flavonoids				
Cat	Qinghai	45.70 ± 26.25 a	33.10 ± 2.40 a	78.81 ± 28.17 a
	Tibet	38.57 ± 0.35 b	32.76 ± 1.83 a	71.33 ± 1.48 b
	Gansu	nd	26.12 ± 4.70 b	26.12 ± 4.70 c
Nar	Qinghai	2.16 ± 0.70 b	9.43 ± 4.96 b	11.59 ± 3.37 b
	Tibet	nd	8.05 ± 0.43 c	8.05 ± 0.43 c
	Gansu	4.44 ± 4.05 a	30.00 ± 27.62 a	34.44 ± 30.56 a
Hes	Qinghai	0.85 ± 0.25 b	97.33 ± 66.50 b	98.18 ± 65.34 b
	Tibet	nd	47.75 ± 10.45 c	47.75 ± 10.45 c
	Gansu	6.07 ± 1.52 a	149.29 ± 11.22 a	155.36 ± 12.07 a
Myr	Qinghai	nd	7.90 ± 1.12 b	7.90 ± 1.12 b
	Tibet	nd	8.06 ± 0.76 b	8.06 ± 0.76 b
	Gansu	nd	36.40 ± 11.24 a	36.40 ± 11.24 a
Que	Qinghai	nd	53.00 ± 20.27 a	53.00 ± 20.27 a
	Tibet	nd	31.73 ± 7.41 b	31.73 ± 7.41 b
	Gansu	nd	32.08 ± 5.60 b	32.08 ± 5.60 b
Nari	Qinghai	7.24 ± 2.85 b	123.19 ± 65.02 b	127.86 ± 65.72 b
	Tibet	6.33 ± 0.01 c	41.49 ± 12.11 c	47.82 ± 12.10 c
	Gansu	11.88 ± 4.88 a	200.25 ± 61.33 a	212.13 ± 56.45 a
Kae	Qinghai	10.69 ± 7.42 b	47.55 ± 10.97 b	58.24 ± 10.06 b
	Tibet	14.18 ± 0.13 a	45.91 ± 23.91 c	60.08 ± 23.77 a
	Gansu	nd	51.33 ± 18.66 a	51.33 ± 18.66 c
Rut	Qinghai	9.90 ± 1.74 c	65.37 ± 16.94 b	75.27 ± 18.26 b
	Tibet	13.29 ± 0.11 a	68.76 ± 12.18 a	82.05 ± 12.06 a
	Gansu	12.07 ± 4.66 b	34.31 ± 10.46 c	46.38 ± 18.47 c
Total	Qinghai	76.54 ± 15.24 a	436.87 ± 40.32 b	510.85 ± 40.78 b
	Tibet	72.37 ± 13.37 b	284.51 ± 20.45 c	356.87 ± 27.29 c
	Gansu	34.46 ± 5.28 c	559.78 ± 66.52 a	594.24 ± 69.70 a

^a^ Phl, phloroglucinol; Gal, gallic acid; Pro, protocatechuic acid; Chl, chlorogenic acid; Dih, 2,4-Dihydroxybenzoic acid; Van, vanillic; Syr, syringic acid; pCo, *p*-coumaric; Fer, ferulic acid; Sal, salicylic acid; Ben, benzoic acid; oCo, *o*-coumaric acid; Dim, 3,4-Dimethoxybenzoic acid; Cat, catechin; Nar, naringin; Hes, hesperidin; Myr, myricetin; Que, quercetin; Nari, naringenin; Kae, kaempferol; Rut, rutin. ^b^ Means in the same line with different letters are significantly different (*p* < 0.05) between the free and bound content of phenolic acid and flavonoids. nd, not detected.

**Table 5:** Analysis of the correlation of the composition and free phenolic content with antioxidant activity.

	FP	FF	TPA	DPPH	FRAP	ABTS
FP	1	0.712 *	0.457	0.821 **	0.711 **	0.789 **
FV	0.712 **	1	0.186	0.395	0.349	0.444
TPA	0.457	0.186	1	0.255	0.592 *	0.355
Gal	−0.465	−0.251	0.065	−0.555	−0.184	−0.583 *
Dih	0.459	0.017	0.344	0.640*	0.104	0.725 **
Pro	0.506	0.246	0.668 *	0.436	0.225	0.648 *
Cat	0.621 *	0.296	0.314	0.493	0.375	0.440
Nari	−0.590 *	−0.477	0.022	−0.355	−0.615 *	−0.143
DPPH	0.821 **	0.395	0.255	1	0.458	0.890 **
FRAP	0.711 **	0.349	0.592*	0.458	1	0.325
ABTS	0.789 *	0.444	0.355	0.890 **	0.325	1

The correlation coefficients was calculated according to the Pearson method; ** indicates significance *p* < 0.01, * indicates significance *p* < 0.05; FP, free phenolic; FF, free flavonoid; DPPH, DPPH^•^ scavenging activity; FRAP, FRAP antioxidant activity ; ABTS, ABTS^•+^ scavenging activity; TPA, total phenolic acid; Gal, gallic acid; Dih, 2,4-dihydroxybenzoic acid; Pro, protocatechuic acid; Cat, catechin; Nari, naringenin.

**Table 6:** Analysis of the correlation of the bound phenolic composition and content with the antioxidant activity.

	BP	BF	TPA	DPPH	FRAP	ABTS
BP	1	0.303	−0.152	0.212	0.718 **	0.576 **
BF	0.303	1	0.303	0.364	0.198	0.485 *
Phl	−0.052	0.327	0.465*	0.155	0.052	−0.017
Chl	0.359	0.200	0.240	0.200	0.141	0.479 *
Van	0.449	0.270	0.180	0.135	0.249	0.539 *
Pco	−0.121	0.273	0.667 **	0.182	−0.290	0.061
Fer	0.606 *	0.333	−0.182	0.001	0.443 *	0.545 *
Dim	−0.242	0.091	0.364	0.121	−0.504 *	−0.364
Hes	−0.303	−0.091	0.364	−0.242	−0.260	−0.485 *
Myr	−0.364	−0.091	0.545 *	−0.182	−0.412	−0.303
Que	0.515 *	0.424	0.152	0.455 *	0.351	0.758 **
Nari	0.311	0.023	0.626 **	0.088	0.086	0.302
Rut	0.516 *	0.121	−0.152	0.273	0.351	0.576 **
DPPH	0.212	0.364	0.152	1	0.107	0.455 *
FRAP	0.718 **	0.198	−0.198	0.107	1	0.412
ABTS	0.576 **	0.485 *	0.091	0.455 *	0.412	1

The correlation coefficients was calculated according to the Pearson method; ** indicates significance *p* < 0.01, * indicates significance *p* < 0.05; BP, bound phenolic ; BF, bound flavonoid; DPPH, DPPH^•^ scavenging activity; FRAP, FRAP antioxidant activity ; ABTS, ABTS^•+^ scavenging activity; TPA, total phenolic acid; Phl, phloroglucinol; Chl, chlorogenic acid; Van, vanillic; Pco, *p*-coumaric; Fer, ferulic acid; Dim, 3,4-dimethoxybenzoic acid; Hes, hesperidin; Myr, myricetin; Que, quercetin; Nari, naringenin; Rut, rutin.
